# Application of Offshore Visibility Forecast Based on Temporal Convolutional Network and Transfer Learning

**DOI:** 10.1155/2020/8882279

**Published:** 2020-10-20

**Authors:** Zhenyu Lu, Cheng Zheng, Tingya Yang

**Affiliations:** ^1^School of Artificial Intelligence, Nanjing University of Information Science and Technology, Nanjing, China; ^2^Jiangsu Collaborative Innovation Center on Atmospheric Environment and Equipment, Nanjing, China; ^3^School of Electronic and Information Engineering, Nanjing University of Information Science and Technology, Nanjing, China; ^4^Jiangsu Meteorological Observatory, Nanjing, China

## Abstract

Visibility forecasting in offshore areas faces the problems of low observational data and complex weather. This paper proposes an intelligent prediction method of offshore visibility based on temporal convolutional network (TCN) and transfer learning to solve the problem. First, preprocess the visibility data sets of the source and target domains to improve the quality of the data. Then, build a model based on temporal convolutional network and transfer learning (TCN_TL) to learn the visibility data of the source domain. Finally, after transferring the knowledge learned from a large amount of data in the source domain, the model learns the small data set in the target domain. After completing the training, the model data of the European Mid-Range Weather Forecast Center (ECMWF) meteorological field were selected to test the model performance. The method proposed in this paper has achieved relatively good results in the visibility forecast of Qiongzhou Strait. Taking Haikou Station in the spring and winter of 2018 as an example, the forecast error is significantly lower than that before the transfer learning, and the forecast score is increased by 0.11 within the 0-1 km level and the 24 h forecast period. Compared with the CUACE forecast results, the forecast error of TCN_TL is smaller than that of the former, and the TS score is improved by 0.16. The results show that under the condition of small data sets, transfer learning improves the prediction performance of the model, and TCN_TL performs better than other deep learning methods and CUACE.

## 1. Introduction

Atmospheric visibility is an indicator used to judge the transparency of the atmosphere. It refers to the maximum horizontal distance that the person with normal vision can distinguish the outline of the target from the background when observing the black target with the sky as the background under the weather conditions at that time [1]. There are many climatic factors that affect the visibility of offshore waters, such as fog, haze, smoke, dust, precipitation, etc. The most important factor is fog [2, 3]. Low visibility often affects the travel safety of offshore vessels and can easily cause accidents at sea. Therefore, how to predict the occurrence of low-visibility weather in offshore areas as much as possible is a problem that researchers are concerned about. Here, the offshore visibility prediction method based on transfer learning proposed in this paper aims to improve the current situation of offshore visibility prediction.

The visibility forecast used to rely on traditional numerical forecasting methods. The weather conditions in the future were calculated through numerical forecasting methods based on the theoretical basis of fluid mechanics, atmospheric dynamics, and thermodynamics. In recent years, with the continuous development and improvement of machine learning and deep learning, the application of machine learning and deep learning to visibility prediction has also become a hot spot for researchers [4–6].

As a commonly used meteorological forecasting method, traditional numerical forecasting is widely used. The National Center for Atmospheric Research (NCAR) has jointly developed a multiscale, multiprocess model system that is online and fully coupled. It is a widely used meteorological–chemical coupled model named weather research and forecast model coupled with chemistry (WRF-Chem) [7, 8]. The US Environmental Protection Agency (EPA) has also developed a universal multiscale air quality model named congestion mitigation and air quality (CMAQ) [9, 10]. These two forecasting models have been localized in various regions in China, and the WRF-Chem model has been introduced in East China to establish a numerical forecast model named Beijing regional environmental meteorology prediction system (BREMPS) in East China. It is applied to visibility prediction in East China [11, 12]. The introduction of the CMAQ model in the southern region established the Southern China regional environmental meteorological numerical forecasting model named Guangdong Regional Assimilation Chemistry Environmental System (GRACES), which was applied to the operational forecasting of the Pearl River Delta [9]. At the same time, the domestic environmental meteorological numerical model is also continuously researched. The Chinese Academy of Meteorological Sciences has independently developed a national-level numerical haze forecasting system named CMA Unified Atmosphere Chemistry Environment (CUACE). CUACE online coupled with the chemical weather model, which has been used in environmental meteorological operations nationwide [13, 14].

In recent years, researchers have achieved a lot of results in machine learning and deep learning [15–19] and have applied them to various fields [20–24]. Among them, machine learning and deep learning also show advantages in weather forecasting such as visibility forecasting and air quality forecasting [25–28]. Machine learning algorithms have excellent performance in visibility forecasting. The Support Vector Machine (SVM) method was used to select multiple kernel functions for the forecasting modeling experiment of low-visibility weather in Shuangliu Airport and study the impact of various meteorological elements on visibility [29]. Long short-term memory-fully connected (LSTM-FC) neural network was used to predict the pollutants in Beijing area and achieved good results [30]. Li et al. used a hybrid CNN-LSTM model developed by combining the convolutional neural network (CNN) with the long short-term memory (LSTM) neural network for forecasting the next 24 h PM2.5 concentration in Beijing [31]. BP neural network was used to construct the visibility forecast model of the Bohai Rim city to reduce the visibility forecast error [32]. In terms of visibility prediction for offshore waters, people used classification and tree regression to establish a forecast model of sea fog along the coast of Qingdao [33]. However, these methods are not ideal for forecasting offshore visibility. They can only predict the general trend of visibility. The accuracy of low-visibility forecasting and visibility-level forecasting needs to be improved.

Both machine learning and deep learning network models are inseparable from large amounts of data. Using deep learning to predict visibility requires a large amount of meteorological data to train the network. However, offshore observation sites are scarce, so using deep learning to face offshore visibility forecasting is faced with the problem of insufficient data. Therefore, this article aims to use transfer learning to solve this problem.

Transfer learning is a machine learning method and a problem-solving idea [34]. In recent years, it has been widely used in various research fields to solve the difficulties caused by insufficient data and achieved excellent performance in building energy prediction and text prediction [35–37]. It is a way to achieve the knowledge transfer between the source and target domains by modeling the distribution of data in the source and target domains, thereby improving the performance of the algorithm [38]. The transfer learning method can better solve the problem of poor forecast performance caused by less data.

In order to improve the forecast accuracy of offshore visibility under the condition of a small data set, this paper uses time convolutional network (TCN) and transfer learning to establish a forecast model to achieve an objective forecast of visibility in offshore areas such as Qiongzhou Strait. At the same time, it improves the ability of forecasting and early warning services for the haze and sea fog in the strait, provides technical support for the low-visibility weather forecast in this area, and ensures the safety of ships traveling.

## 2. Materials and Methods

### 2.1. Data Source and Data Preprocessing

**Figure 1:**
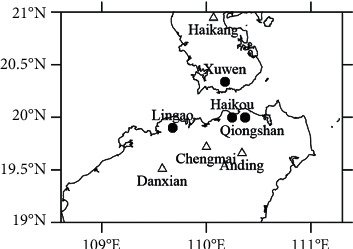
Distribution of representative sites of Qiongzhou Strait.

The climatic conditions in the coastal area of South China are subtropical monsoon climate, which belongs to the East Asian monsoon region. The meteorological background is greatly affected by relative humidity, and sea fog occurs frequently in winter and spring, so low-visibility weather occurs more frequently. The training data used in this article is divided into two parts. The first part is the meteorological data and environmental data of the stations in Leizhou Peninsula and northern Hainan, which is the source domain data. The second part is a small amount of meteorological data and environmental protection data of the weather stations on both sides of the Qiongzhou Strait, which is the target domain data. The specific data of the first part include routine ground observation data and high-altitude data of Leizhou Peninsula area and northern Hainan area from 2016 to 2018, such as data on wind speed, relative humidity, temperature, pollutants, etc., and visibility observation data. The specific data of the second part include visibility observation data and meteorological data and environmental protection data of the offshore sites of the Qiongzhou Straits on both sides of the north and south sides of the Qiongzhou Strait from January to April 2016–2018. The verification data used in this paper is the European Meteorological Forecast Center (ECMWF) meteorological field model data in 2018. In this paper, four inland sites are selected as source domain site data, namely, Haikang (59750), Danxian (59845), Chengmai (59843), and Anding (59851). The data of four stations on the seashore were selected to conduct a forecast experiment on the visibility of Qiongzhou Strait, namely, Xu Wen (59754), Haikou (59758), Lingao (59842), and Qiongshan (59757).  Figure 1 shows the distribution of the eight sites.

Because the weather forecasting factors are composed of different parts, it is necessary to compose the data for time series analysis and time-space matching and then compose the input data that meets the requirements. At the same time, due to various factors in the real-time observation of the meteorological conditions by the meteorological station, the meteorological observation data will often have some observations missing and abnormal, so the data should be cleaned. This paper uses Lagrange interpolation to fill in missing values. Lagrange interpolation formula is

(1) $$\begin{array}{c} y=a_{0}+a_{1}x+a_{2}x^{2}+\cdots +a_{n-1}x^{n-1}, \end{array}$$

(2) $$\begin{array}{c} L\left(x\right)=y_{1}\frac{\left(x-x_{2}\right)\left(x-x_{3}\right)\cdots \left(x-x_{n}\right)}{\left(x_{1}-x_{2}\right)\left(x_{1}-x_{3}\right)\cdots \left(x_{1}-x_{n}\right)}+y_{2}\frac{\left(x-x_{2}\right)\left(x-x_{3}\right)\cdots \left(x-x_{n}\right)}{\left(x_{2}-x_{1}\right)\left(x_{2}-x_{3}\right)\cdots \left(x_{2}-x_{n}\right)}+\cdots y_{n}\frac{\left(x-x_{2}\right)\left(x-x_{3}\right)\cdots \left(x-x_{n}\right)}{\left(x_{n}-x_{1}\right)\left(x_{n}-x_{3}\right)\cdots \left(x_{n}-x_{n-1}\right)}. \end{array}$$

Equation (2) solves the Lagrange interpolation polynomial *L*(*x*) and then substitutes the point corresponding to the missing value *x* to obtain the approximate value of the missing value.

The visibility data sample is composed of two parts, meteorological data and environmental protection data, which have a large number of features. In order to improve efficiency, the characteristics of the data samples need to be screened. In this paper, the Pearson correlation coefficient method is used to measure the degree of correlation between two variables. The Pearson correlation coefficient method is used to calculate the correlation between the features that affect visibility and the observation of visibility, and the features are screened by comparing the correlations of the various impact features. The formula of the correlation coefficient is shown in formula (3). In this paper, *X* represented the meteorological feature in the data, and *Y* represented the value of visibility:

(3) $$\begin{array}{c} \rho_{XY}=\frac{\mathrm{cov}\left(X,Y\right)}{\sigma_{X}\sigma_{Y}}=\frac{E\left[\left(X-\mu x\right)\left(Y-\mu y\right)\right]}{\sigma_{X}\sigma_{Y}}. \end{array}$$

**Figure 2:**
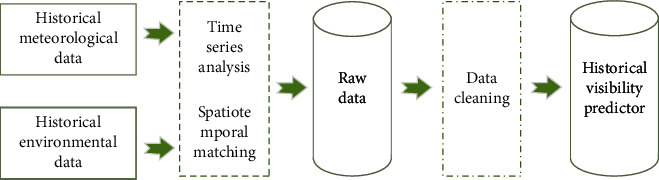
Data preprocessing flowchart.

The forecast meteorological elements of visibility in the data include 40 different forecast meteorological elements such as temperature, relative humidity, wind shear, and wind speed. The meteorological elements of visibility forecast after screening by the Pearson coefficient method are shown in Table 1. The visibility forecast meteorological elements screened by the Pearson coefficient method are shown in Table 1. In this paper, 12 meteorological elements are selected and input into the network as forecast factors. Table 1 shows the correlation coefficients between each forecasting factor and the observation of visibility. It can be clearly observed that the correlation coefficients of temperature and humidity are relatively high. Visibility in Haikou area is highly correlated with temperature, relative humidity, etc., which is a good proof of the reliability of using the correlation coefficient method to filter visibility prediction factors here [39]. The forecast feature data includes historical weather data and historical environmental data, which are matched in time and space to form the original visibility data. The raw data are cleaned to obtain the final visibility forecast data. The processing flow is shown in Figure 2.

### 2.2. Method

#### 2.2.1. Temporal Convolutional Network

Because the Temporal Convolutional Network (TCN) is a network structure that can better handle time series data, this paper uses TCN to build a prediction model of the visibility of the source domain. TCN is the second architecture that can analyze temporal data in addition to the Recurrent Neural Network (RNN) architecture. The structure of TCN is shown in Figure 2. TCN has two main characteristics: First, there is a causal relationship between the layers of the convolutional network, which means that there will be no “bobble” historical information or future data. Even if a long short-term memory network (LSTM) with the same time series processing function has a memory gate, it cannot completely remember all the historical information, let alone if some information is useless in the LSTM will gradually be forgotten [40]. Second, architecture of TCN can be flexibly adjusted to any length, and it can be mapped to correspond to several interfaces according to the output terminal. This is the same as the RNN framework, which is very convenient. The TCN network adopts the form of convolution, which is mainly composed of causal convolution, hole convolution, residual module, and full convolution network. Among them, causal convolution is used to make the network suitable for sequence models. The output of the convolution layer at time *t* is only convolved with the elements of the current layer and the previous layer. The causal convolution calculation formula at *x*_*t*_ is

(4) $$\begin{array}{c} \left(F*X\right)\left(x_{t}\right)=\sum_{k=1}^{K}f_{k}x_{t-K+k}. \end{array}$$

Among them, filter is *F*=(*f*_1_, *f*_2_,…, *f*_*k*_), and input sequence is *X*=(*x*_1_, *x*_2_,…, *x*_*T*_). The use of the hole convolution and residual modules allows the TCN network to remember history. The hole convolution kernel is

**Figure 3:**
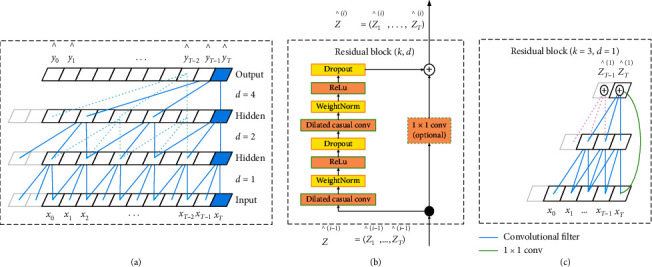
TCN architecture diagram. (a) Skip connection in TCN residual module. (b) Residual module of TCN. (c) Skip connection in TCN residual module.

(5) Fs=x∗fds=∑i=0k−1fi·Xs−d·i,

where *d* is the hole coefficient, *k* is the size of the filter, and *s* − *d* · *i* is the past direction [40]. The structure of the hollow convolution is shown in Figure 3(a). When the filter is *F*=(*f*_1_, *f*_2_,…, *f*_*k*_), input sequence is *X*=(*x*_1_, *x*_2_,…, *x*_*T*_), and the convolution of the holes at *x*_*t*_ is

(6) F∗Xdxt=∑k=1Kfkxt−K−kd.

In formula (6), *d*can increase the receptive field, and the size is (*K* − 1)*d*+1. The formula for the residual module is

(7) $$\begin{array}{c} o=\text{activation}\left(x+ℱ\left(x\right)\right). \end{array}$$

In formula (7), ℱis a part of a series of transformations, and *x* is an input. The structure diagrams are shown in Figures 3(b) and 3(c).

Full convolutional networks make the output and input dimensions consistent, simplifying the network. It is these parts that make up TCN, which makes the network have the advantages of good parallelism, flexible receptive fields, small training memory, and adjustable input sequence length compared with LSTM.

#### 2.2.2. Transfer Learning

Time convolutional networks belong to deep neural networks. During the training process of deep neural networks, the network will generate a large number of network parameters, so a large amount of data is needed to train the parameters. However, there is a problem of low data volume in offshore visibility, and the learning effect is not good. Therefore, the method of transfer learning is used to solve this problem.

**Figure 4:**
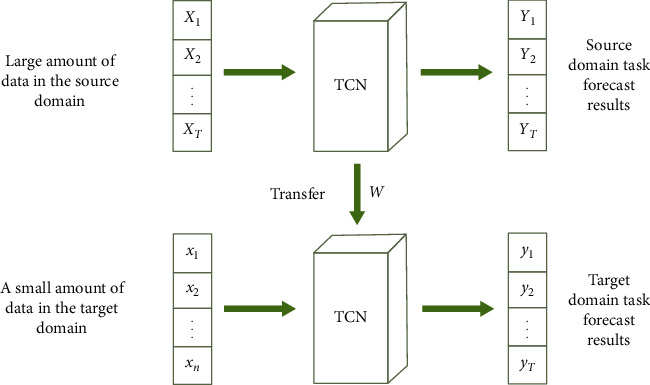
The transfer process of the source domain model to the target domain model.

However, there is a problem of low data volume in offshore visibility, and the learning effect is not good. Therefore, the method of transfer learning is used to solve this problem. Researcher defined the transfer learning in detail: given source domain *D*_*S*_={(*X*_1_^(*S*)^, *Y*_1_^(*S*)^),…, (*X*_*n*_*s*__^(*S*)^, *Y*_*n*_*s*__^(*S*)^)} and source domain learning tasks *T*_*s*_, as well as target domain *D*_*t*_={*X*_1_^(*t*)^,…, *X*_*n*_*t*__^(*t*)^} and target domain learning tasks *T*_*t*_. The ultimate goal of transfer learning is to gradually improve the performance of the prediction function in the target domain *D*_*S*_ and the target domain task *T*_*s*_ by learning the knowledge in the source domain *D*_*t*_ and the source domain task *T*_*s*_ [41]. In this paper, a prediction model is established in the source domain task, and a large amount of source domain data is used as the training data of the model, and the pretrained model is saved after training. Transfer learning is used to make the target domain task network inherit the weights of the pretrained model. When performing a new task, a small amount of new visibility data is used as input, and the weight of the pretrained model loaded into the source domain task is used as the initial weight of the target domain network for training. The training process is shown in Figure 4.

### 2.3. Evaluation Method

From January to April 2018, there was frequent sea fog in the Qiongzhou Strait area with low visibility. The classification of visibility observation data during this period is relatively clear, and the selection of data during this period can make the experiment comprehensive effect better.

In this paper, there are two ways to evaluate the forecast performance, which are numerical test and classification test. The numerical test uses two indicators: root mean square error (RMSE) and mean absolute error (MAE). RMSE is used to measure the deviation between the observed value and the predicted value. It usually reflects the precision of the measurement well. The smaller the value of RMSE, the higher the precision. MAE represents the average value of the absolute error between the predicted value and the observed value. The smaller the value of MAE, the higher the accuracy of prediction. MAE represents the average value of the absolute error between the predicted value and the observed value. The smaller the value of MAE, the higher the accuracy of prediction. The calculation formula of the two is as follows:

(8) $$\begin{array}{c} \text{RMSE}\left(X,P,O\right)=\sqrt{\frac{1}{m}\overset{m}{\underset{i=1}{\sum }}{\left(p\left(x_{i}\right)-o_{i}\right)}^{2}}, \end{array}$$

(9) $$\begin{array}{c} \text{MAE}\left(X,P,O\right)=\frac{1}{m}\sum_{i=1}^{m}\left|p\left(x_{i}\right)-o_{i}\right|. \end{array}$$

In formulas (8) and (9), *m* represents the number of samples, *X* represents the forecast factor of historical visibility, *P* represents the predicted value of the model for visibility, and *O* represents the observed value of historical visibility.

In this paper, the visibility level is mainly divided into four levels, namely, 0∼1 km, 1∼5 km, 5∼10 km, and above 10 km. Among them, improving the forecast accuracy of the visibility level of 0 to 1 km has important practical guiding significance. The graded forecast test uses TS scoring rules to test forecast performance of the model for each visibility level. The formula for the test method is as follows:

(10) $$\begin{array}{c} \text{TS}=\frac{\text{NA}}{\text{NA}+\text{NB}+\text{NC}}. \end{array}$$

In the formula, NA represents the number of correct forecasts, NB represents the number of empty forecasts, and NC represents the number of missed forecasts. Correct forecast means that the forecast level is the same as the live level; empty forecast means that the forecast level is less than the live level; and missed forecast means that the forecast level is higher than the live level.

### 2.4. Forecasting Process

**Figure 5:**
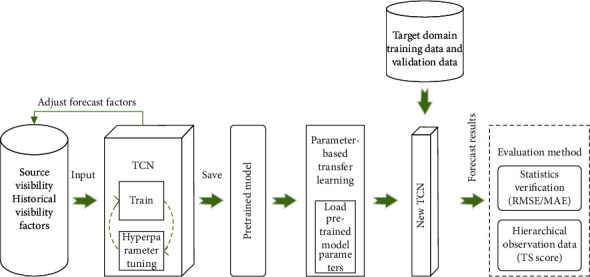
Technical process of visibility forecast based on TCN and transfer learning.

The forecast technology flow used in this paper is shown in Figure 5. This forecast model uses the TCN model and transfer learning method to forecast the Qiongzhou Strait. In the first step, the source domain prediction factor obtained after data preprocessing is used as the input feature of the TCN network to train the network. Based on the loss function result, adjust the parameters to optimize the model performance iteratively and save the optimal training model. In the second step, the transfer learning method based on parameter transfer is used to transfer the weight of the pretrained model in the source domain as the initial weight of the network and start learning the data in the target domain. Finally, input the EWMCF meteorological field data of the forecast period to check the model performance and output the forecast results.

## 3. Results and Discussion

### 3.1. Lab Environment

The experiment in this paper is implemented on TensorFlow 1.14.0 framework under Ubuntu 16.04 system and uses GPU to accelerate. The hardware configuration of the experimental platform is CPU: Intel Core i5-8600k, GPU: NVIDIA GTX 1080Ti, and memory is 16G.

### 3.2. Experimental Parameter Setting

Hyperparameters are the parameters set by the network model before learning, not the parameters obtained through training. Hyperparameter settings are crucial to the efficiency of model learning. Under normal circumstances, the model training should be selected before the start of learning. After observing the value of the loss function and the training status during the model learning process and adjusting the hyperparameters, the model learning efficiency is the highest. Among many hyperparameters, hyperparameters such as learning rate and batch size have the greatest impact on the efficiency and accuracy of learning. After repeated experiments, the model performs best when the learning rate is 0.002, the batch size is 60, and the hidden unit is 150.

### 3.3. Result Analysis

#### 3.3.1. Comparison of Results before and after Transfer Learning

**Figure 6:**
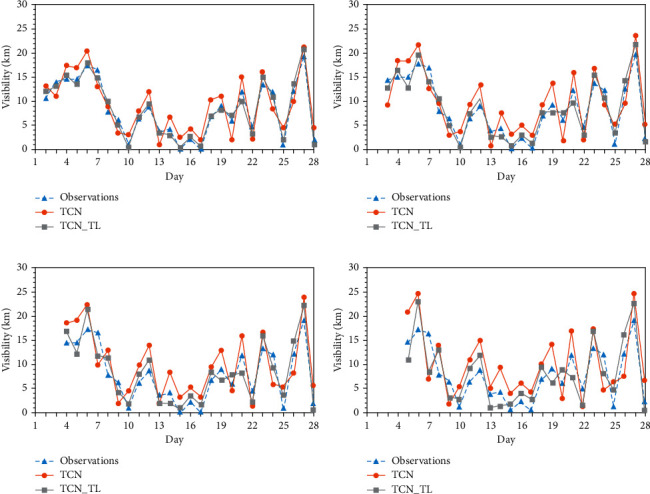
Daily changes of the visibility observation and forecast results of (a) 24 h, (b) 48 h, (c) 72 h, and (d) 96 h at the Haikou Station in February 2018.

After establishing the prediction model named TCN_TL based on TCN and transfer learning, the network was tested and evaluated by using the offshore visibility data from January to April 2018.  Figure 6 shows the MAE and RMSE of the forecasted visibility values of Haikou Station before and after the transfer learning in February 2018, including 24 h, 48 h, 72 h, and 96 h four-time error comparison.  Figure 6 takes the Haikou Observation Station in February 2018 as an example and gives the visibility observation values and forecast values that change daily. It is not difficult to find that in each forecast period, the visibility before and after transfer learning can better reflect the change trend of visibility, but there is a deviation between the two in the forecast value and magnitude.  Table 2 shows the graded forecast score of Haikou Station in February 2018, named TS score. By comparing the RMSE and MAE of different forecast aging and the grading forecast scores of different aging, we can find that, regardless of migration, the error of short-term forecast is always smaller than the error of long-term forecast. And the TS score of short-term forecasts is always higher than the longer time-sensitive TS score. Therefore, it can be concluded that the performance of short-term forecast is better than that of long-term forecast.

**Figure 7:**
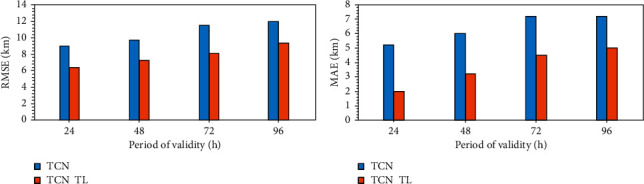
Statistical characteristics between the observed and forecasted visibility of February 2018 at Haikou Station. (a) RMSE. (b) MAE.

Taking the 24-hour time-effect forecast as an example, the visibility forecast of the Haikou Station before and after transfer learning in February 2018 is analyzed.  Figure 7 shows the forecast situation before and after transfer learning. The forecast value of TCN_TL for visibility is closer to the actual value of visibility than the forecast value of TCN. After transfer learning, both RMSE and MAE decreased significantly, RMSE decreased from 9 km to 6.2 km, and MAE decreased from 5.2 km to 2 km. As shown in Table 2, the accuracy of the grading forecast has also improved. At the 0∼1 km level, the TS score increased from 0.23 to 0.35. At the 1∼5 km level, the TS score increased from 0.41 to 0.5, and at the 5∼10 km level, the TS score was 0.52, increased to 0.67. And the accuracy rate increased from 0.64 to 0.76 at the 10∼35 km level. It is worth noting that although the TS score has increased in all levels, there is still room for improvement. Through transfer learning, the match between the predicted value of visibility and the observed value has been significantly improved.

#### 3.3.2. Comparison and Analysis of Different Model Results

**Figure 8:**
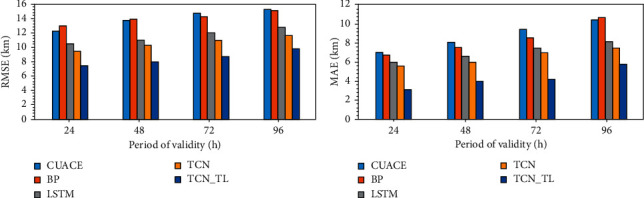
Statistical characteristics between the observed and forecasted visibility from February to April 2018 at Haikou Station. (a) RMSE. (b) MAE.

In order to better test the prediction performance of TCN_TL for visibility in offshore areas, this paper compares its experimental results with the experimental results of the other three models without transfer learning. It should be noted that the following experiments used historical visibility forecast data from 2016 to 2018 to forecast the visibility from January to April 2018 in the Qiongzhou Strait region.  Figure 8 shows the errors between the predicted and observed values for the next 24 h, 48 h, 72 h, and 96 h under different models. The forecast errors of each model are given in the figure, which are the TCN_TL and CUACE models, the forecast model based on BP neural network, the forecast model based on LSTM network, and the forecast model based on TCN. Among them, the TCN_TL model uses two parts of the historical visibility observation data of the source domain and the target domain during training, while other models use the historical visibility observation data of the target domain for training.

From the data shown in Figure 8, it can be found that no matter what kind of forecast model, the longer the forecast time, the greater the error between the model's forecast and the observed value. Among them, the performance of the forecast model based on TCN_TL is significantly better than other forecast models.  Figure 8(a) shows the root-mean-square error of the predicted and observed visibility of the model. Taking the 24 hr forecast period of validity as an example, the RMSE of the CUACE model is higher than other models. The LSTM network prediction model has a lower RMSE than the BP neural network, while the TCN prediction model has a slightly lower RMSE than LSTM. The RMSE of the TCN_TL prediction model using the transfer learning method is significantly lower than the RMSE of the TCN prediction model and other prediction models. The RMSE of the five forecasting models is 14.2 km, 14 km, 10.5 km, 9.4 km, and 7.4 km, respectively.  Figure 8(b) shows the average absolute error between the predicted value and the observed value of the model. The MAE of the five forecasting models is 7 km, 6.7 km, 6 km, 5.6 km, and 3.1 km.

From the analysis in Figure 8, we can see that TCN_TL using the transfer learning method performs better than the other four models in both RMSE and MAE. Comparing TCN with the CUACE model, the BP neural network prediction model, and the LSTM prediction model, the performance of TCN is relatively good. From the comparison of the errors between the TCN and TCN_TL models, the use of transfer learning to compensate for the problem of small data volume significantly reduces the error between the predicted visibility and the observed visibility and improves the prediction performance of the model.

Generally speaking, the weather with low-visibility level, especially the weather with a level of 0∼1 km, appears less frequently than ordinary weather, and the opportunities for model learning are also much less. It is relatively low, but improving the accuracy of this level of forecasting has practical guiding significance. As shown in Table 3, taking the 24 hr forecast as an example, when the visibility is less than 1 km, the BP neural network prediction model is only 0.18. The TS score of TCN is slightly higher than LSTM, reaching 0.25. TCN_TL has the highest score, reaching 0.36. In addition, at the other three levels, the TS score of TCN_TL is also higher than the other four forecast models. Therefore, the results show that, compared with other traditional methods and machine learning methods, TCN_TL has more advantages in predicting the performance of offshore visibility.

### 3.4. Visibility Spatial Distribution Analysis

**Figure 9:**
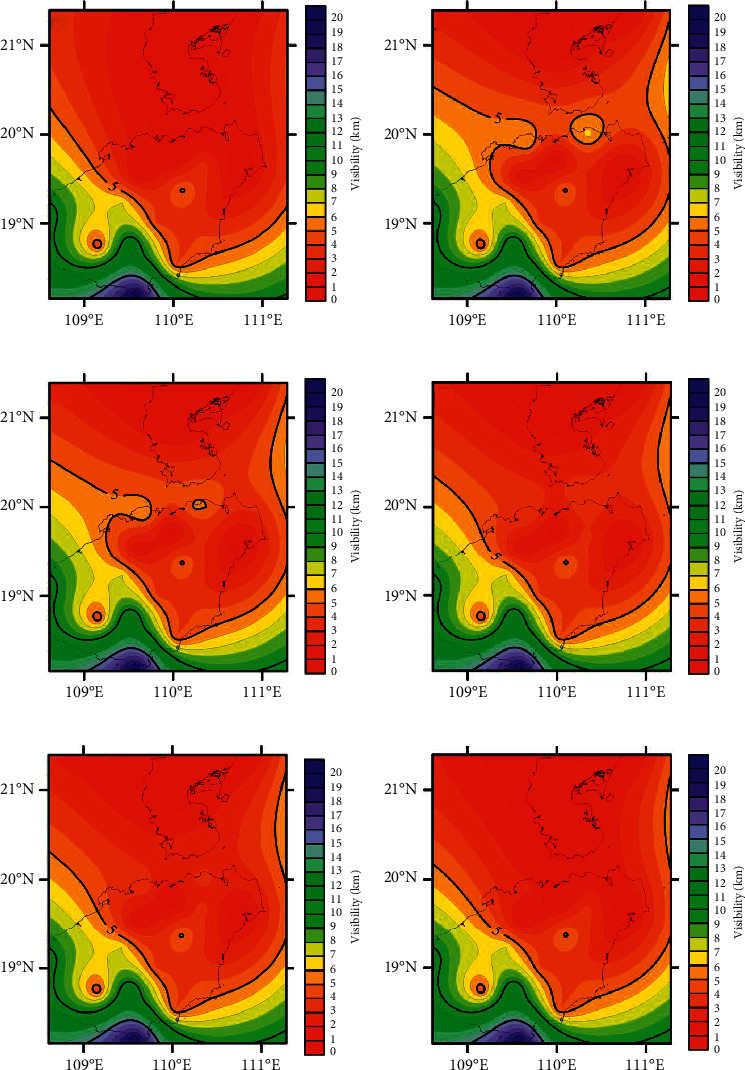
Visibility spatial distribution. (a) February 16th 8:00 measured map. (b) February 16th 8:00 CUACE model forecast map. (c) February 16th 8:00 BP model forecast map. (d) February 16th 8:00 LSTM model forecast map. (e) February 16th 8:00 TCN model forecast map. (f) February 16th 8:00 TCN_TL model forecast map.

During the Spring Festival of 2018, the Qiongzhou Strait experienced persistent low-visibility weather. This section takes this event as an example and uses a 24 hr forecast model to perform forecast analysis.  Figure 9 shows the visibility forecast of each model at 8 AM on February 16. Because the Kriging interpolation method considers the variation distribution of spatial attributes, it can effectively eliminate the errors caused by uneven sampling and make the results more in line with the actual situation [42]. Here, the Kriging interpolation method is used to interpolate the visibility spatial results of the Qiongzhou Strait, which better shows the spatial distribution of visibility. According to the spatial distribution of actual observation results of medium visibility in Figure 9(a), the Qiongzhou Strait is under low-visibility weather. Comparing the spatial distribution of visibility prediction results in Figures 9(b)–9(f), the overall spatial distribution state is gradually tending towards the actual spatial distribution state of visibility and the prediction result space of TCN_TL. The distribution is closest to the spatial distribution of actual observations. Comparing Figures 9(e) and 9(f), it can be found that the prediction result of TCN_TL is closer to the actual observation result than the prediction result of TCN. Therefore, the use of transfer learning improves the prediction accuracy of the model.  Figure 9(b) is the spatial distribution of the CUACE forecast results. The forecast results are higher than the actual observation values, and the TCN_TL forecast results are more in line with the actual situation. Therefore, under the condition of small data set, the prediction performance of TCN_TL is better than that of CUACE.

## 4. Conclusions

The offshore visibility prediction method based on transfer learning proposed in this paper combines TCN and transfer learning. This paper uses TCN to establish a source domain forecast model and learns the knowledge of the source domain under the premise that the source domain has a large amount of data. And TCN_TL was used to forecast the visibility of the target domain offshore. The following conclusions can be obtained through experimental analysis:

1. This paper compares the results of TCN and TCN_TL for forecasting offshore visibility. The experimental results show that the model of transfer learning can be used to learn the visibility knowledge of the source domain under the condition of a small amount of offshore meteorological observation data to improve the accuracy of the visibility forecast of the target domain.
2. In this paper, the TCN network is used to learn the source domain data. Compared with LSTM and BP neural networks, the RMSE and MAE between TCN prediction and observation are smaller, and the TS score is relatively higher than others' in each visibility forecast level. Therefore, TCN is more advantageous for learning and predicting time series like visibility data than others.
3. The 24-hour forecast of offshore visibility of Haikou Station from January to April 2018 was taken as an example. Under the conditions of small data sets, comparing the forecast results of TCN_TL and CUACE, the forecast error of TCN_TL is lower than that of CUACE, and the RMSE and the MAE are 6.8 km and 3.9 km. RMSE drops to 7.4 km, and MAE drops to 3.1 km. TS score of TCN_TL has also improved in each forecast level. At the level of 0∼1 km, the TS score is 0.36, increased by 0.16. At the level of 1∼5 km, the TS score is 0.52, increased by 0.19. At the level of 1∼5 km, the TS score is 0.52, increased by 0.19. At the level of 5∼10 km, the TS score is 0.67, increased by 0.29. At the level of greater than 10 km, the TS score is 0.78, increased by 0.39. Therefore, under the condition of a small data set, TCN_TL has an advantage in predicting the visibility of offshore waters than CUACE.
4. It is worth noting that, compared with the different aging prediction of each model, no matter which model, the prediction error of the short aging is lower than that of the longer aging. Therefore, the model method proposed in this paper is more suitable for the short aging prediction of the offshore visibility.

## Figures and Tables

**Table 1:** Forecast factors and correlation coefficients.

Predictor	Pearson's correlation coefficient absolute value
Temperature	0.563
925 hPa temperature	0.511
900 hPa temperature	0.498
950 hPa horizontal wind speed	0.489
Atmospheric pressure	0.473
925 hPa horizontal wind speed	0.397
Vertical wind speed	0.390
925 hPa relative humidity	0.379
900 hPa horizontal wind speed	0.372
900 hPa relative humidity	0.338
Depression of the dew point	0.333
Relative humidity	0.328
950 hPa vertical wind speed	0.323

**Table 2:** TS scores of visibility forecast in February 2018 at Haikou Station.

Classification (km)	0∼24 h		24∼48 h		48∼72 h		72∼96 h	
	TCN	TCN_TL	TCN	TCN_TL	TCN	TCN_TL	TCN	TCN_TL
[0, 1]	0.23	0.35	0.15	0.26	0.1	0.2	0.1	0.15
[1, 5]	0.41	0.5	0.36	0.54	0.3	0.45	0.23	0.31
[5, 10]	0.52	0.67	0.5	0.61	0.42	0.56	0.51	0.58
[10, 35]	0.64	0.76	0.6	0.7	0.56	0.7	0.63	0.63

**Table 3:** 24 hr time-efficient grading forecast TS score.

Classification (km)	CUACE	BP	LSTM	TCN	TCN_TL
[0, 1]	0.14	0.18	0.21	0.25	0.36
[1, 5]	0.33	0.35	0.4	0.45	0.52
[5, 10]	0.38	0.37	0.51	0.58	0.67
[10, 35]	0.47	0.48	0.62	0.68	0.78

## Data Availability

The data of this research work can be found through open data set. The ECMWF model data were obtained from the ECMWF website (https://www.ecmwf.int./en/forecasts/datasets/browse-reanalysis-datasets). The daily visibility data and the CUACE model data were provided by the National Meteorological Center.
